# Is predictive coding theory articulated enough to be testable?

**DOI:** 10.3389/fncom.2015.00111

**Published:** 2015-09-08

**Authors:** Naoki Kogo, Chris Trengove

**Affiliations:** Laboratory of Experimental Psychology, Brain and Cognition, University of Leuven (KU Leuven)Leuven, Belgium

**Keywords:** predictive coding, visual cortex, feedback, physiological, generative model, bayesian models, neuroanatomy, error signals

Predictive coding theory (Srinivasan et al., 1982; Mumford, 1992; Rao and Ballard, 1999) claims that the function of the hierarchical organization in the cortex is to reconcile representations and predictions of sensory input at multiple levels. It does this because the dynamics of neural activity is geared toward minimizing the *error*: the difference between the input representation at each level and the prediction originating from a higher level representation. In other words, the neural activities in the whole hierarchy settle to a state where the difference between the prediction and the representation of sensory input is minimal. This view has gained enormous popularity, and research applying this theoretical framework to explain various kinds of empirical data has flourished since then (Friston, 2010; Clark, 2013b).

Predictive coding theory is a mechanistic theory: it aims to describe the neurocomputational machinery. Hence, merely describing phenomenological data in the terminology of the theoretical framework is not sufficient. The theory should allow the empirical data to be explained by neurocomputational mechanisms and the proposed mechanisms should be testable at the neurophysiological level. To do this, the details of the mechanisms, especially how the errors are computed and minimized, need to be articulated in neuronal terms. Note that error signals at each level influence neural activities in two ways in this framework. First, they are fed forward to the higher level(s) where they influence the neural activities of the higher level representation(s). The resulting predictions are in turn fed back to the lower level. Second, at the same time, the error signals also influence the response properties of the neurons at the same level and the representation of the sensory input is modified. The updating of the prediction and the changes to the lower level representation are made to improve their match. Through this two-way process of reconciliation the error signals are minimized. However, the possibility of simultaneous changes in both higher level prediction and lower level representations, and mixed populations of error neurons and sensory representation neurons within the same local circuit, give rise to a “multiple choices” problem. This problem is significant when using empirical data such as fMRI, EEG, and unit recordings to test the theory. For example, how do we determine whether a single unit being recorded is an error neuron or a neuron representing input? Note that in the model of Rao and Ballard (1999), endstopping cells function as error neurons because they signal the sudden stop of the line segment while continuation of the line is predicted. On the contrary, Kapadia et al. (1995) showed that the neural response to a line segment increases when collinear line segments are presented outside of the classic receptive field. In the former case, the increase of the neural signal is explained because of the mismatch of the input with the prediction while in the latter case, the increase would be explained because the input matches the prediction. How is this apparent inconsistency of explanations resolved? Or consider Kanizsa's illusory surface (Kanizsa, 1955). It has been shown that neurons in lower level visual cortex are activated at the location of illusory contours (von der Heydt et al., 1984). Are they considered as error neurons or representation neurons? In other words, are they active because of the mismatch between the input and the prediction giving rise to error signal, or because the representation signal is modified to match the prediction? The same applies to recordings of the activity of a population of neurons such as those obtained via fMRI: is an increase of the fMRI signal due to an increased error signal or to changes in the input representation? And does the process of reconciliation between the lower level representation and the prediction result in silencing of error neurons and if so, is this detectable in the data? The last question is particularly crucial because it has been suggested that reduction of neural signals at the lower level can be explained in terms of error minimization (Murray et al., 2002; Summerfield et al., 2008; den Ouden et al., 2009; Alink et al., 2010; Todorovic et al., 2011; Kok et al., 2012b). To overcome these problems of testability, the theory must be articulated in sufficient neurophysiological detail, particularly in regard to the mechanisms of error computation and minimization.

Predictive coding theory is inspired by a systematic pattern of connectivity, both within individual areas of neocortex and within the feedforward and feedback projections between areas, specific to layer location and type of source and target neurons (Maunsell and van Essen, 1983). These anatomical patterns suggest that neurocomputational processes are based on a characteristic neural circuit comprising intra-areal and inter-areal connections, and that this neural circuit as a module is iterated in a hierarchical fashion. The iterated circuit block that Rao and Ballard (1999) proposed is an example of this “canonical microcircuit,” an elementary neural circuit that is constructed in a specific way and works as a principal module of the computation. The next step toward testability is to specify how the proposed neural computation is accomplished using more realistic cortical neurons and circuitry. A paper by Bastos et al. (2012) addressed this very issue by first presenting the set of equations that implement the dynamics of predictive coding and then matching the terms in the equations to the neural sub-types in the different layers. However, the neurocomputational mechanisms to realize predictive coding theory are still in debate (den Ouden et al., 2012; Eriksson et al., 2012; Gotts et al., 2012; Kok et al., 2012a; Clark, 2013a,b; Rauss and Pourtois, 2013; Summerfield and de Lange, 2014). In this paper, through the analysis of the logic behind the Bastos model, we raise some issues in regard to the critical question for the predictive coding theory: what, in neuronal terms, is an error signal and how is it computed? We consider this question as a central issue of the predictive coding theory.

Their point of departure is the generative model: an iterative and centrifugal sequence of “causes” (*v*) and “states” (*x*). The cause in the parent level (*i* + 1) creates the state in the child level (*i*), which in turn becomes the parent level of the next child level (*i*−1). Then, they created a feedback system by introducing bi-directional interactions between the modules. The conditional expectation of state and cause and their errors are computed at each level of the hierarchy, the error signals are sent to the higher level, and the expectation signals are sent to the lower level. The expectations and the errors for both causes and states are denoted by μ and ξ respectively (their Equation 1). Hence, there are four main variables per level, μ_*v*_, μ_*x*_, ξ_*v*_, and ξ_*x*_. (Each of these variables is multi-dimensional, according to the dimensionality of the input representation at each level.)

By analysing the sequential processes in Equation 1 and the known neural types and their connections in neocortex, they pointed out the “remarkable correspondence” between the sequential processes in the equations and the neural architecture. Accordingly, they proposed a mapping between the processes in Equation 1 and a neural microcircuit (their Figure 5), according to which distinct neuron sub-types function as the terms μ_*v*_, μ_*x*_, ξ_*v*_, and ξ_*x*_.

The operation of the circuit is as follows:

1. The prediction signal, *g*(*i* + 1) at level *i* + 1 is created as a function of μ_*v*_(*i* + 1) and μ_*x*_(*i* + 1) at layer 5/6 and is sent to the lower level (*i*).
2. At the lower level, the error signal, ξ_*v*_(*i* + 1), is computed at layer 2/3 by comparing *g*(*i* + 1) with μ_*v*_(*i*).
3. The error signal, ξ_*v*_, is sent to the layer 4 of the higher level via feedforward connections (and re-represented by the excitatory neurons at that layer).
4. The error signal of state, ξ_*x*_(*i*), in layer 4 is updated according to the expectations of cause and state at the same level.
5. The error signals help to update the expectations of cause and state (μ_*v*_ and μ_*x*_) by modifying the excitatory neurons at layer 2/3.
6. The expectations of cause and state (μ_*v*_ and μ_*x*_) are re-represented at layer 5/6 to create the prediction signal, *g* to be sent to the lower level (step 1).

In the proposed framework, the error is the difference between the lower level representation and the prediction. Hence, the error is,

error= “representation” minus “prediction”

This corresponds to the error computation occurring in the superficial layer (step 2), subtracting *g*(*i* + 1) from μ_*v*_(*i*). This formulation appears to cause some problems.

In their model, the feedback signal, *g*, is sent from the layer 5/6 neurons (μ_*v*_ and μ_*x*_) at the higher level. These are excitatory cells. It is, then, not clear how the subtraction can be made when this signal reaches the superficial layer at the lower level. Note that while the feedback signal sent from the higher level is *g* (Figure 1A corresponding to their Figure 5 right; at bottom), when it reaches the top layer at the lower level, it is *-g* (Figure 1B corresponding to their Figure 5 right; at top) without any explanation of the reversal of the sign. Although they suggested the involvement of inhibitory neurons in L1 earlier, among the diversity of distinct types of inhibitory neurons (Petilla Interneuron Nomenclature Group et al., 2008) many of them can “provide strong mono-synaptic inhibition to L2/3” (page 699) and there are no clear reasons given why the L1 inhibitory neurons should take the role of reversing the sign of *g*. Furthermore, they did not explicitly specify the function of the sign reversal by inhibitory neurons in Figure 5. Moreover, they also pointed out that (page 699) “feedback connections can both facilitate and suppress firing in lower hierarchical areas.” How can this dualistic effect be exhibited by this circuit? Note that certain formulations of predictive coding have been shown to be functionally equivalent to a biased competition framework (Spratling, 2008) in which the error signal is computed within the upper level rather than at the lower level. Therefore, it may be possible, that with the different mapping of variables to neuronal sub-types, the biologically implausible top-down inhibition for subtraction is avoided.

**Figure 1:**
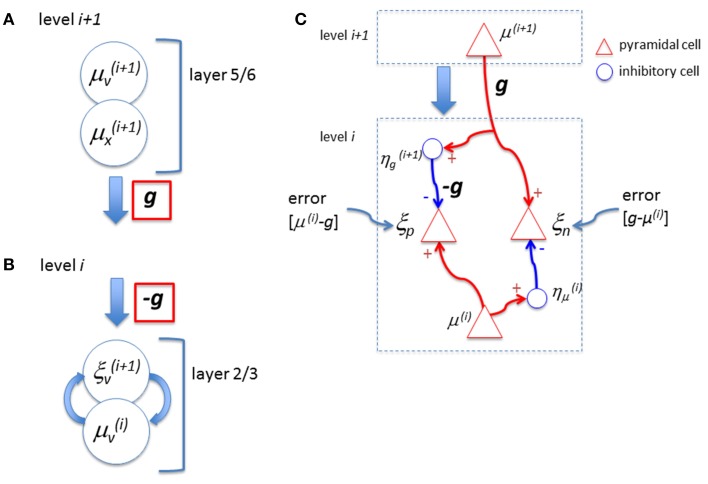
**(A)** Bastos et al. (2012) proposed that neurons in layer 6 represent expectation of cause, *μ*_*v*_, and expectation of state, *μ*_*x*_, which send out feedback signals to the lower level. In their diagram, this output signal is expressed as a function *g* (red). **(B)** When this signal arrives at the lower level, the feedback signal is expressed as −*g* (red) in their proposal without any explanation of the reversal of the sign. Note that, to compute the error, the subtraction is done between the lower level representation signal, *μ*_*v*_, and the prediction factor *g*, (*ξ*_*v*_*= μ*_*v*_ − *g*) and, hence, the negative signal of *g* is necessary. However, if the neurons, *μ*_*v*_ and *μ*_*x*_ are pyramidal (excitatory) cells as proposed by Bastos et al. this subtraction cannot be performed. **(C)** The error, “representation – feedback,” can create either positive or negative values. However, the neuron that represents the error in the proposed circuit of Bastos et al. would not create action potentials when the error value is negative. Hence, the neuron is not capable to signal the error when the prediction factor *g* is larger than the representation signal, *μ*_*v*_. To deal with the positive and negative error signals properly, “two distinct populations of neurons to signal errors, one for positive and another for negative errors” (Rao and Ballard, 1999) may be necessary. For example, the inhibitory neuron, *η*${}_{\mu }^{\left(i\right)}$, shown here reverses the sign of the feedforward representation signal, *μ*^(*i*)^, to compute the “negative” error, *ξ*_*n*_ (=*g*−*μ*^(*i*)^). The other inhibitory neuron, *η*${}_{g}^{\left(i+1\right)}$, reverses the sign of *g* so that the “positive” error can be expressed as a neural signal in *ξ*_*p*_ (=*μ*^(*i*)^ − *g*).

Next, consider how the error signal is represented. Assume that the prediction signal fed back to the lower level is stronger than the representation signal. As their definition of the error is “*representation minus prediction*,” the error value becomes negative. However, they claim that the error neuron, ξ_*v*_, is a pyramidal cell and, hence, ξ_*v*_(*i* + 1) in their Figure 5 is always excitatory. In other words, this circuit cannot create an explicit “negative signal” that is sent to the higher level. There could be two ways to solve this problem. One way to signal the “negativity” is to assume that there is a baseline level of activity in ξ_*v*_(*i* + 1) and the negativity is expressed by the *decrease* of the output signal ξ_*v*_(*i* + 1) below the baseline. If this is the case, the error is minimized the most when the activities of the error neurons reach the baseline state, not when they become silent. Having a certain level of baseline activity means that the energy consumption by the error neuron is not necessarily minimal when the error is minimized. This is quite a different view to that of minimizing (or silencing) the activity of error neurons, even though the latter view is a central component of predictive coding theory. For example, Friston (2005) wrote, “High-level predictions explain away prediction error and tell the error units to ‘shut up’.” (p. 829), and Kok et al. (2012a) wrote, “high-level predictions explain away prediction error, thus silencing error neurons” (p. 265). A second way to signal negativity, which retains the concept of minimizing error neuron activity, is a neural circuit with more explicit error computation to deal with positive and negative errors (Figure 1C). Note that Rao and Ballard (1999) suggested the possibility of such computation of positive and negative errors (p. 85). If this is the case, the proposed neural circuit by Bastos et al. is not an explicit representation of how the error computation is achieved in the real biological system. Alternatively, it has been suggested that error minimization can be done by a divisive operation (Koch and Poggio, 1999) which might avoid the need for negative error signals. However, this requires the equilibrium state to be represented by baseline activities, which leads to the same problem discussed above.

Intra-areal microcircuits and their inter-areal bi-directional connections in cortex follow a systematic, recurring pattern that suggests a hierarchically iterated canonical signal processing. How exactly these circuits process information is an outstanding question of great importance. Predictive coding theory is currently a highly influential theory for cognitive function and behavior, and one of the plausible theoretical frameworks that may explain the signal processing architecture of the cortex. A “translation” of the terms in the mathematical formulation of the theory into neurophysiological and neuroanatomical parameters would have a strong impact on the precise design of experiments involving neural recordings and psychophysics. The analysis of the neurocomputational model by Bastos et al. presented here suggests that the way in which error signals are computed is the central issue for testing the theory, and that there is still a gap between the theoretical formalism and concrete neural mechanisms.

## Conflict of interest statement

The authors declare that the research was conducted in the absence of any commercial or financial relationships that could be construed as a potential conflict of interest.
